# Association of Polymorphisms of the Receptor for Advanced Glycation Endproducts Gene with Schizophrenia in a Han Chinese Population

**DOI:** 10.1155/2017/6379639

**Published:** 2017-03-08

**Authors:** Jiawu Fu, Xiang Zuo, Jingwen Yin, Xudong Luo, Zheng Li, Juda Lin, Bin Zhao, Guoda Ma, Keshen Li

**Affiliations:** ^1^Department of Neurology, Affiliated Hospital of Guangdong Medical University, Zhanjiang, Guangdong 524001, China; ^2^Institute of Neurology, Affiliated Hospital of Guangdong Medical University, Zhanjiang, Guangdong 524001, China; ^3^Department of Psychiatry, Affiliated Hospital of Guangdong Medical University, Zhanjiang 524001, China; ^4^Unit on Synapse Development and Plasticity, National Institute of Mental Health, National Institutes of Health, Bethesda, MD, USA; ^5^Stroke Center, Neurology & Neurosurgery Division, The Clinical Medicine Research Institute & The First Affiliated Hospital, Jinan University, Guangzhou, China

## Abstract

Receptor for Advanced Glycation Endproducts (RAGE) is a member of the immunoglobulin superfamily that binds diverse ligands involved in the development of inflammatory damage and diverse chronic diseases including schizophrenia. Here, three single-nucleotide polymorphisms (SNPs) (G82S, -374T/A, and -429T/C) in the* RAGE* gene were genotyped in 923 patients with schizophrenia and 874 healthy-matched controls in a Han Chinese population using the SNaPshot technique. Additionally, we investigated the association among aforementioned SNPs with the clinical psychotic symptoms of the patients and neurocognitive function. Our study demonstrated that the frequencies of the TC + CC genotypes and the C allele in the -429T/C polymorphism were significantly lower in the patients compared with the controls (*p* = 0.031 and *p* = 0.034, resp.). However, the significant effect disappeared when using Bonferroni correction (*p* = 0.093 and *p* = 0.102, resp.). And there were no significant differences in the genotype and allele frequencies between the patients and the controls for G82S and -374T/A polymorphisms. Additionally, the -429T/C C allele carriers had marginally higher Symbol coding scores than the subjects with the TT genotypes [*p* = 0.031 and *p* (corr) = 0.093]. Our data indicate that the* RAGE* -429T/C polymorphism may be associated with the susceptibility of schizophrenia.

## 1. Introduction

Schizophrenia is a complex psychiatric disorder which is characterized by distortion of thought, emotion, and behavior, with a lifetime risk of approximately 1% [1]. In recent years, neuroinflammatory responses, such as increases in proinflammatory mediators interleukin-6 (IL-6) and tumour necrosis factor-*α* (TNF-*α*), have been demonstrated to be involved in the pathology of schizophrenia [2]. Numerous studies have reported the strongest signal for schizophrenia in the genetic region (chromosome 6p22.1) which is closely related to the major histocompatibility complex (MHC) [3]. Based on these, many genes involved in the inflammatory processes may be considered to be associated with schizophrenia.

Receptor for Advanced Glycation Endproducts (RAGE) is a member of the immunoglobulin superfamily of cell surface molecules capable of interacting with diverse ligands, including advanced glycation endproducts (AGEs), high-mobility group box-1 proteins (HMGB1), and S100/calgranulins [4]. Recent studies have reported that the interaction of RAGE with S100B expressed on glial cells and neurons resulted in the activation of nuclear factor-*κ*B (NF-*κ*B), which in turn induced the secretion of the proinflammatory mediators IL-6 and TNF-*α* [5, 6]. Steiner et al. [7] showed that a reduction of S100B levels during reconvalescence from acute schizophrenia was regulated by its scavenger soluble Receptor for Advanced Glycation Endproducts (sRAGE), which was hypothesized to counteract the detrimental action of RAGE as a decoy receptor of the signaling pathway. Recently, decreased levels of circulating sRAGE have been found in the patients with schizophrenia, suggesting a loss of sRAGE or imbalance of RAGE/sRAGE as a potential pathogenetic mechanism in the development of schizophrenia [8]. Taken together, these observations make the gene encoding RAGE a logical candidate gene for schizophrenia.

The gene encoding RAGE, comprising eleven exons, is located on chromosome 6p21.3 near the major histocompatibility complex locus [9]. To date, the* RAGE* gene has been identified having several single-nucleotide polymorphisms (SNPs) including G82S (rs2070600), -374T/A (rs1800624), and -429T/C (rs1800625), which may have marked effects on the expression or function of RAGE. The -374T/A and -429T/C polymorphisms in the promoter region of the* RAGE* gene were shown to exert functional effects on the basal transcriptional activity, and the G82S polymorphism in the exon 3 of the* RAGE* gene displayed enhanced ligand-binding affinity and influenced the generation of proinflammatory mediators [10, 11]. A recent European case-control study presented evidence for an association between the G82S polymorphism in the* RAGE* gene and schizophrenia [12]. However, studies regarding the association of other polymorphisms in the* RAGE* gene with schizophrenia have not been previously investigated. Moreover, no study has hitherto investigated* RAGE* gene polymorphisms in the Han Chinese population. Based on the potential role of RAGE in the pathogenetic mechanism of schizophrenia, the aim of this study was to examine the potential association between the G82S, -374T/A, and -429T/C polymorphisms in the* RAGE* gene and schizophrenia in a Han Chinese population.

## 2. Materials and Methods

### 2.1. Subjects

The present study consecutively recruited 923 unrelated patients with schizophrenia (587 males and 336 females; mean age = 35.56 ± 13.79 years) and 874 healthy controls (528 males and 346 females; mean age = 36.47 ± 14.45 years) from the Department of Psychology and the Health Examination Center at the First Affiliated Hospital of Guangdong Medical University, respectively. The healthy controls were well matched for age and gender with the patients with schizophrenia (*p* = 0.170 and 0.164, resp.). All the aforementioned subjects were Han Chinese in origin. The patients with schizophrenia were diagnosed by at least two experienced senior psychiatrists on the basis of the Diagnostic and Statistical Manual of Mental Disorders IV (DSM-IV) criteria for schizophrenia. Meanwhile, the clinical psychotic symptoms of the patients with schizophrenia were evaluated using the Positive and Negative Syndrome Scale (PANSS) [13]. None of the patients with schizophrenia had histories of neurological disorders, organic brain damage, mental retardation, drug or alcohol abuse, metabolic illnesses, and autoimmune disorders. Additionally, all healthy controls lacked family histories (first-degree relatives) of psychiatric disorders, substance abuse diagnosis (excluding nicotine dependence), and serious somatic disorders. The present study was performed with the approval of the Ethics Committee of the First Affiliated Hospital of Guangdong Medical University. All participants provide informed consent, and the patients' capacity to consent was confirmed by the direct relatives when required.

### 2.2. Genotyping

Genomic DNA from EDTA-treated peripheral blood was extracted using the TIANamp Blood DNA Kit (Tiangen Biotech, Beijing, China). A total of 1797 individuals were genotyped for the G82S, -374T/A, and -429T/C polymorphisms located in the* RAGE* gene using the SNaPshot technique (Applied Biosystems, Foster City, CA, USA) as described previously [14]. The polymerase chain reaction (PCR) primers used for the G82S polymorphism were 5′-GCTGGGGTTGAAGGCTTTTTCT-3′ (forward primer) and 5′-CCGGACAGAAGCTTGGAAGGTC-3′ (reverse primer), and the primers used for the -374T/A and -429T/C polymorphisms were 5′-CCCATCTTGATTGCGCAAAGTT-3′ and 5′-TCAAAAAACATGAGAAACCCCAGAA-3′.

### 2.3. Neurocognitive Function Assessment

The neurocognitive function of the patients with schizophrenia was evaluated using the Brief Assessment of Cognition in Schizophrenia (BACS) [15]. The BACS consisted of brief assessments of the following six aspects of cognition: working memory (digit sequencing task), verbal fluency (category instances and controlled oral word association test), verbal memory (list learning), motor speed (token motor task), reasoning and problem solving (Tower of London), and attention and processing speed (Symbol coding).

### 2.4. Statistical Analyses

The normally distributed quantitative data were expressed as mean ± standard deviations (SD) and were compared using Student's *t*-tests between the two different groups. The genotype and allele distributions in different groups were assessed with Pearson's Chi-square test or Fisher's exact two-tailed test. The Hardy-Weinberg Equilibrium (HWE) in the patients and the controls was checked using Pearson's Chi-square test. Bonferroni correction for multiple testing was applied by the number of SNPs. All the statistical analyses were performed using SPSS 19.0 software for Windows, and the criterion for statistical significance was defined as *p* < 0.05. Power calculations were performed using the QUANTO 1.2 software (University of Southern California, LA, USA).

In addition, the linkage disequilibrium (LD) status of the G82S, -374T/A, and -429T/C polymorphisms was determined using the Haploview 4.2 program. Only those haplotypes with frequencies greater than 3% were further analyzed.

## 3. Results

### 3.1. Association Study of SNPs (G82S, -374T/A, and -429T/C) and Schizophrenia

The distribution of genotypes of the three SNPs (G82S, -374T/A, and -429T/C) reached the HWE in both the patients with schizophrenia and the controls (all *p* > 0.05). Allele and genotype frequencies for the* RAGE* gene are shown in Table 1. A significant difference was found between the patients with schizophrenia and the controls in the distribution of the -429T/C polymorphism. The respective frequencies of TT, TC, and CC genotypes were 87.4%, 12.2%, and 0.3% in the patients with schizophrenia and 83.9%, 15.7%, and 0.5% in the controls. The prevalence of the TC + CC genotypes (C allele carriers) and C allele frequencies was significantly lower in the patients with schizophrenia than in the controls (12.6% versus 16.1%, *p* = 0.031, odds ratio [OR] = 0.747, 95% confidence interval [CI] = 0.573–0.974 and 6.4% versus 8.3%, *p* = 0.034, OR = 0.762, 95% CI = 0.592–0.980, resp.). Nevertheless, no statistically significant differences were observed after Bonferroni correction [*p* (corr) = 0.093 and *p* (corr) = 0.102, resp.]. In addition, the male patients with schizophrenia showed marginally lower TC + CC genotypes and C allele frequencies compared to the controls (*p* = 0.036, OR = 0.697, 95% CI = 0.497–0.978 and *p* = 0.054, OR = 0.731, 95% CI = 0.531–1.007, resp.) (Table 2). However, for the G82S and -374T/A polymorphisms, there were no significant differences in the genotype and allele frequencies between the patients with schizophrenia and the controls (Table 1). Moreover, in the gender-stratified analysis, no significant differences were detected between the patients with schizophrenia and the controls (Table 2).

Power analysis revealed that, with our study sample and assuming a risk allele frequency of 8.3%, we would have 95.3% power to detect a genotype relative risk with an odds ratio of 1.5 at the 0.05 level. However, the power was 12.6% for an odds ratio of 1.1 at a significance level of 0.05.

For further analysis, our study also focused on the description of relevant clinical characteristics of 804 patients with schizophrenia distributed by genotype. None of the different genotypes of the three SNPs showed significant associations with age at onset, duration of illness, PANSS total score, and family psychotic history (Table 3). However, for the G82S polymorphism, the presence of the S variant was found to be associated with marginally higher scores in the P subscore compared with the carriers of the GG genotype [*p* = 0.041 and *p* (corr) = 0.123]. Additionally, for the -374T/A polymorphism, the subjects with the TA + AA genotypes exhibited marginally higher P subscore in comparison with the subjects with the TT genotype [*p* = 0.043 and *p* (corr) = 0.129].

### 3.2. Linkage Disequilibrium Analysis and Haplotype Analysis

We also performed a haplotype analysis based on the three SNPs (G82S, -374T/A, and -429T/C). Four out of five possible haplotypes revealed at least 3% frequencies in either the patients with schizophrenia or the controls. Analysis of the haplotype frequencies demonstrated no significant difference between the patients with schizophrenia and the controls (Table 4).

### 3.3. Neurocognitive Function Analysis

The sample for the neurocognitive function analysis using BACS consisted of 205 clinically stable patients with schizophrenia. No significant differences were found in the scores of the seven BACS items among the different genotypes of the G82S or -374T/A polymorphism. However, the -429T/C C allele carriers had marginally higher Symbol coding scores than the subjects with the TT genotypes [*p* = 0.031 and *p* (corr) = 0.093] (Table 5).

## 4. Discussion

Increasing evidences have supported inflammation may act as critical driving force in the pathology of schizophrenia [16]. In line with the proinflammatory actions of the S100B-RAGE axis, strong evidence is implicating elevated serum and CSF levels of S100B in the psychopathology of schizophrenia. Further, the negative symptoms of the patients with schizophrenia have been found to correlate with increasing S100B serum levels [17, 18]. It has been reported that S100B exerts toxic effects on neurons in schizophrenia by inducing neuronal apoptosis via mechanisms involving RAGE-dependent overproduction of neuroinflammatory mediators. Additionally, sRAGE has been recently suggested to regulate the detrimental effects of S100B observed in the patients with schizophrenia [7]. Moreover, a recent case-control study of the Caucasian population identified the G82S polymorphism in the* RAGE* gene as a possible susceptibility locus for schizophrenia [12]. On the basis of these observations, it is reasonable to speculate that the* RAGE* gene might be a logical candidate involved in the underlying cause of schizophrenia.

In the present study, we have evaluated the potential association of the three polymorphisms (G82S, -374T/A, and -429T/C) of the* RAGE* gene with schizophrenia. To the best of our knowledge, this is the first study investigating the association of the* RAGE* genetic polymorphisms with schizophrenia in a Han Chinese population. The results of the present study provide evidence that a genetic variant in the promoter of the* RAGE* gene (-429T/C) may be associated with the risk of developing schizophrenia, in contrast with the G82S and -374T/A polymorphisms. Some evidences have showed that the* RAGE* gene polymorphisms could alter the expression or the function of RAGE, thus affecting the development of various diseases [10, 11]. The -429T/C polymorphism, in the promoter of the* RAGE* gene, has an obvious effect on transcriptional activity [19]. In this study, the TC + CC genotypes and the C allele in the patients with schizophrenia are marginally lower than controls. It indicated that the C allele of the -429T/C polymorphism may be a protective factor of schizophrenia. Interestingly, these results are in line with the observation of Zeng et al. who suggested that the C allele had significantly lower* RAGE* transcriptional activity compared with the T allele in human U937 cells [19]. Based on these findings, we speculate that the T > C variation may downregulate RAGE expression via altering gene transcriptional activity, thereby contributing to protective roles in schizophrenia. Additionally, our study demonstrated that the carriers with the -429T/C C allele had marginally higher Symbol coding scores for attention compared with the subjects with the TT genotypes [*p* = 0.031 and* p* (corr) = 0.093]. It is believed that the patients with schizophrenia commonly demonstrate a compromised neurocognitive profile, and the facets of cognition identified as impaired in the patients with schizophrenia mainly include attention, working memory, and social cognition [20]. With the present findings in mind, it is probable that the -429T/C polymorphism influences the cognitive function of the patients with schizophrenia.

The G82S polymorphism is located in exon 3 that codes for the ligand-binding site; it is possible that the observed effect of the S allele is associated with enhanced ligand binding, consequently increasing the downward receptor signaling [11]. Previous study has shown an association between the G82S polymorphism of the RAGE gene and the risk of schizophrenia in the Caucasian population. However, our data showed no significant differences in the genotype and allele frequencies between schizophrenia and controls in the Han Chinese population. The S allele frequency of the G82S polymorphism was higher in our Chinese population than the corresponding frequency in the Sweden (22.1% versus 3.1%) but was similar to those in the Northern Chinese population (http://hapmap.ncbi.nlm.nih.gov/). The distinction of these results might be due to the genetic heterogeneity in different cohorts, and this finding still needs to be replicated. Additionally, our study found that the* RAGE* G82S S allele carriers have marginally higher positive symptom scores than GG genotype carriers [21.92 ± 7.50 versus 20.84 ± 7.30, *p* = 0.041 and* p* (corr) = 0.123]. Interestingly, previous study has shown that the* RAGE* G82S S allele carriers have significantly higher scores for the psychoticism personality trait including suspicion subscales, which is included among the positive symptoms of schizophrenia [12]. It is conceivable that the* RAGE* gene polymorphisms may be associated with the positive symptoms and may serve as foundational evidence in developing more detailed phenotypic symptomology of schizophrenia.

In relation to the -374T/A polymorphism, in vitro experiments by Hudson et al. [10] demonstrated that the A allele resulted in a threefold increase in transcriptional activity compared with the T allele, which may alter RAGE-ligands interaction leading to altered downstream signaling cascade and consequently contributing to the etiology of diabetic vascular complications. However, no significant differences were found in the genotype and allele frequencies between schizophrenia and controls in our case-control study. This might be because the* RAGE* gene plays different roles in the pathogenesis of different diseases. Interestingly, we found that the* RAGE* -374T/A A allele carriers have marginally higher scores for the positive symptoms of schizophrenia compared with the TT-homozygote carriers [22.41 ± 7.76 versus 21.16 ± 7.30, *p* = 0.043 and *p* (corr) = 0.129]. The animal experiment has showed that the transgenic mice with the overexpression of S100B would display the deficiency of learning ability and behavior disorder, suggesting that the potential relationship between the S100B and the positive symptoms of schizophrenia [21]. Based on these, we speculate that the -374T/A polymorphism alters the RAGE-S100B interaction leading to the alteration of the S100B level via influencing the transcriptional activity of the* RAGE* gene and thus affecting the positive symptoms of schizophrenia.

Our study has several limitations. First, power analysis revealed that, with our study sample and assuming a risk allele frequency of 8.3%, we would have 95.3% power to detect a genotype relative risk with an odds ratio of 1.5 at the 0.05 level. However, the power was 12.6% for an odds ratio of 1.1 at a significance level of 0.05. Therefore, a false negative result cannot be excluded. Second, our sample size in this study was relatively small, and no statistically significant differences were observed after Bonferroni correction. Furthermore, we did not conduct the study which clarified the relationship between other SNPs in the* RAGE* gene and schizophrenia.

In conclusion, our study is the first to describe the possible existence of an association of the* RAGE* gene polymorphism with the risk of schizophrenia in a Chinese population. Our data revealed that the* RAGE* gene -429T/C polymorphism may be associated with the susceptibility of schizophrenia in Chinese Han population, and the C allele of the -429T/C polymorphism may be a protective factor for schizophrenia.

## Figures and Tables

**Table 1:** Genotype and allele frequency of the *RAGE* gene in the patients with schizophrenia and controls.

dbSNP ID	Schizophrenia	Control	*p* value	OR (95% CI)
	*n* = 923 (%)	*n* = 874 (%)		
G82S				
Genotype				
GG	535 (58.0)	533 (61.0)	0.426^a^	
GS	336 (36.4)	296 (33.9)		
SS	52 (5.6)	45 (5.1)		
GS + SS	388 (42.0)	341 (39.0)	0.192^b^	1.134 (0.939–1.369)
Allele				
G	1406 (76.2)	1362 (77.9)		1.000 (reference)
S	440 (23.8)	386 (22.1)	0.212	1.104 (0.945–1.290)

-374T/A				
Genotype				
TT	704 (76.3)	688 (78.7)	0.341^a^	
TA	199 (21.6)	173 (19.8)		
AA	20 (2.2)	13 (1.5)		
TA + AA	219 (23.7)	186 (21.3)	0.215^c^	1.151 (0.922–1.437)
Allele				
T	1607 (87.1)	1549 (88.6)		1.000 (reference)
A	239 (12.9)	199 (11.4)	0.152	1.158 (0.947–1.415)

-429T/C				
Genotype				
TT	807 (87.4)	733 (83.9)	0.081^a*∗*^	
TC	113 (12.2)	137 (15.7)		
CC	3 (0.3)	4 (0.5)		
TC + CC	116 (12.6)	141 (16.1)	0.031^d^	0.747 (0.573–0.974)
Allele				
T	1727 (93.6)	1603 (91.7)		1.000 (reference)
C	119 (6.4)	145 (8.3)	0.034	0.762 (0.592–0.980)

OR: odds ratio; 95% CI: 95% confidence interval. ^a^Global test for the three different genotypes. ^b^Calculations were performed, GS + SS versus GG. ^c^Calculations were performed, TA + AA versus TT. ^d^Calculations were performed, TC + CC versus TT. ^*∗*^Fisher's exact test.

**Table 2:** Genotype and allele frequency of the *RAGE* gene in the patients with schizophrenia and controls, according to gender.

dbSNP ID	Male		*p* value OR (95% CI)	Female		*p* value OR (95% CI)
	Patient	Control		Patient	Control	
	*n* = 587 (%)	*n* = 528 (%)		*n* = 336 (%)	*n* = 346 (%)	
G82S						
Genotype						
GG	342 (58.3)	328 (62.1)	0.408^a^	193 (57.4)	205 (59.2)	0.798^a^
GS	214 (36.5)	173 (32.8)		122 (36.3)	123 (35.5)	
SS	31 (5.3)	27 (5.1)		21 (6.3)	18 (5.2)	
GS + SS	245 (41.7)	200 (37.9)	0.189^b^	143 (42.6)	141 (40.8)	0.632^b^
Allele						
G	898 (76.5)	829 (78.5)	1.000 (reference)	508 (75.6)	533 (77.0)	1.000 (reference)
S	276 (23.5)	227 (21.5)	0.256 1.122 (0.920–1.370)	164 (24.4)	159 (23.0)	0.535 1.082 (0.843–1.389)

-374T/A						
Genotype						
TT	443 (75.5)	417 (79.0)	0.226^a^	261 (77.7)	271 (78.3)	0.948^a^
TA	133 (22.7)	106 (20.1)		66 (19.6)	67 (19.4)	
AA	11 (1.9)	5 (0.9)		9 (2.7)	8 (2.3)	
TA + AA	144 (24.5)	111 (21.0)	0.164^c^	75 (22.3)	75 (21.7)	0.839^c^
Allele						
T	1019 (86.8)	940 (89.0)	1.000 (reference)	588 (87.5)	609 (88.0)	1.000 (reference)
A	155 (13.2)	116 (11.0)	0.109 1.233 (0.954–1.593)	84 (12.5)	83 (12.0)	0.766 1.048 (0.758–1.449)

-429T/C						
Genotype						
TT	516 (87.9)	441 (83.5)	0.086^a*∗*^	291 (86.6)	292 (84.4)	0.386^a*∗*^
TC	68 (11.6)	85 (16.1)		45 (13.4)	52 (15.0)	
CC	3 (0.5)	2 (0.4)		0 (0.0)	2 (0.6)	
TC + CC	71 (12.1)	87 (16.5)	0.036^d^	45 (13.4)	54 (15.6)	0.412^d^
Allele						
T	1100 (93.7)	967 (91.6)	1.000 (reference)	627 (93.3)	636 (91.9)	1.000 (reference)
C	74 (6.3)	89 (8.4)	0.054 0.731 (0.531–1.007)	45 (6.7)	56 (8.1)	0.325 0.815 (0.542–1.225)

OR: odds ratio; 95% CI: 95% confidence interval. ^a^Global test for the three different genotypes. ^b^Calculations were performed, GS + SS versus GG. ^c^Calculations were performed, TA + AA versus TT. ^d^Calculations were performed, TC + CC versus TT. ^*∗*^Fisher's exact test.

**Table 3:** Clinical characteristics of the patients with schizophrenia and distribution by genotypes of the three SNPs.

Parameters	G82S		*p* value	-374T/A		*p* value	-429T/C		*p* value
	GG	GS + SS		TT	TA + AA		TT	TC + CC	
	*n* = 458	*n* = 346		*n* = 616	*n* = 188		*n* = 703	*n* = 101	
Age at onset (years)	25.27 ± 9.89	25.34 ± 10.31	0.926	25.28 ± 9.93	25.34 ± 10.53	0.946	25.46 ± 10.19	24.17 ± 9.14	0.228
Duration of illness (years)	11.49 ± 9.94	11.26 ± 9.67	0.742	11.53 ± 9.94	10.94 ± 9.44	0.475	11.35 ± 9.77	11.66 ± 10.20	0.768
Years of education (years)	9.30 ± 3.20	9.11 ± 3.24	0.405	9.19 ± 3.22	9.34 ± 3.21	0.58	9.21 ± 3.23	9.33 ± 3.12	0.725
PANSS total score	74.08 ± 17.58	74.66 ± 18.29	0.651	73.85 ± 17.49	76.25 ± 19.45	0.131	74.61 ± 17.91	73.05 ± 18.48	0.416
P subscore	20.84 ± 7.30	21.92 ± 7.50	0.041	21.16 ± 7.30	22.41 ± 7.76	0.043	21.54 ± 7.38	20.85 ± 7.77	0.383
N subscore	17.65 ± 7.86	17.39 ± 8.05	0.648	17.55 ± 8.09	17.36 ± 7.54	0.774	17.53 ± 7.88	17.32 ± 8.54	0.802
G subscore	35.59 ± 9.00	35.35 ± 9.29	0.716	35.14 ± 8.94	36.48 ± 9.82	0.096	35.54 ± 9.09	34.88 ± 9.72	0.502
Family psychotic history	75 (16.4%)	45 (13.0%)	0.184	91 (14.8%)	29 (15.4%)	0.826	106 (15.1%)	14 (13.9%)	0.748
Age at onset (years)									
<18	84 (18.3%)	67 (19.4%)	0.713	115 (18.7%)	36 (19.1%)	0.883	131 (18.6%)	20 (19.8%)	0.779
≥18	374 (81.7%)	279 (80.6%)		501 (81.3%)	152 (80.9%)		572 (81.4%)	81 (80.2%)	

Values are the mean ± SD; PANSS: Positive and Negative Syndrome Scale.

**Table 4:** Haplotype analysis of the three SNPs in the *RAGE* gene in the patients with schizophrenia and controls.

(G82S)-(-374T/A)-(-429T/C)	Schizophrenia *n* (%)	Controls *n* (%)	*p* value	OR (95% CI)
G-T-T	1063.1 (57.6)	1027.4 (58.8)		1.000 (reference)
S-T-T	424.9 (23.0)	376.6 (21.5)	0.305	1.089 (0.925–1.282)
G-A-T	223.9 (12.1)	189.6 (10.8)	0.227	1.139 (0.922–1.407)
G-T-C	119.0 (6.4)	145.0 (8.3)	0.076	0.793 (0.613–1.025)

Haplotype frequency < 0.03 in both schizophrenic patients and controls has been dropped; OR odds ratio; 95% CI 95% confidence interval.

**Table 5:** Neurocognitive functions of the patients with schizophrenia and distribution by genotypes of the three SNPs.

Parameters	G82S		*p* value	-374T/A		*p* value	-429T/C		*p* value
	GG	GS + SS		TT	TA + AA		TT	TC + CC	
	*n* = 121	*n* = 84		*n* = 159	*n* = 46		*n* = 185	*n* = 20	
BACS									
Digit sequencing task	16.64 ± 9.30	14.93 ± 9.71	0.203	16.05 ± 9.70	15.57 ± 8.80	0.761	14.85 ± 8.95	16.06 ± 9.56	0.589
Category instances	30.73 ± 12.75	31.33 ± 12.56	0.737	30.59 ± 13.37	32.30 ± 9.73	0.339	26.30 ± 10.84	31.48 ± 12.75	0.082
Controlled oral word association test	9.86 ± 5.71	10.36 ± 6.50	0.563	10.01 ± 5.95	10.24 ± 6.39	0.823	7.60 ± 4.37	10.33 ± 6.14	0.054
List learning	22.06 ± 12.07	22.19 ± 13.67	0.942	21.62 ± 12.71	23.83 ± 12.73	0.300	22.55 ± 9.77	22.06 ± 13.02	0.872
Token motor task	44.91 ± 9.80	47.31 ± 14.24	0.182	46.03 ± 11.97	45.43 ± 11.54	0.767	47.05 ± 13.88	45.77 ± 11.65	0.647
Tower of London	7.70 ± 4.85	7.62 ± 4.86	0.934	7.47 ± 5.89	8.35 ± 5.18	0.438	5.70 ± 3.79	7.88 ± 4.80	0.169
Symbol coding	20.38 ± 12.52	21.17 ± 12.76	0.661	20.13 ± 12.70	22.67 ± 12.16	0.229	14.95 ± 9.49	21.32 ± 12.75	0.031

Values are the mean ± SD; BACS: Brief Assessment of Cognition in Schizophrenia.
